# The Book World of Medicine and Science

**Published:** 1906-08-18

**Authors:** 


### THE BOOK WORLD OF MEDICINE AND SCIENCE.

THE ANIMAL PARASITES OF MAN, a Handook for Students and Medical Men. By Dr. MAX BRAUN, Professor of Zoology and Comparative Anatomy, Königsberg, Prussia. Third enlarged and improved edition, with 294 illustrations in the text. Translated from the German by PAULINE FALCKE. [Brought up to date by LOUIS W. SAMBON, M.D. (Naples), Lecturer at the London School of Tropical Medicine, etc., and FRED. V. THEOBALD, M.A.] (London: John Bale, Sons and Danielson, Limited, Oxford House, Great Titchfield Street, Oxford Street, W. 1906. All rights reserved. Demy 8vo pp. 453. Price 21s. net.)

TEN years ago only the most advanced students had the slightest notion of the importance of animal parasites in the dissemination of disease. Now, thanks to the labours of recent investigators, chiefly to those devoted workers in tropical fields, parasitology as applied to disease has become a distinct science. The important discoveries recently made have dragged to light many unknown animal parasites exercising a pernicious effect on the health of man their human host. Helminthology, until lately a despised and rejected subject of pathological learning, has acquired an importance no longer to be ignored. Protozoology, formerly hardly referred to in medical works, threatens now to outstrip bacteriology in the brilliancy of its achievements. It is well known that insects and their congeners play such an extensive *rôle* in preventive medicine and in the diffusion of disease as almost to have initiated a new era in the science of medicine. No practitioner alive to the indications of modern progress and methods of research can afford to be without some reliable handbook to guide and help him in this new field. Therefore not only to the investigator and researcher in this new pathological territory, but to the practitioner who has perforce to be content as learner of what is new and useful, a work like Dr. Max Braun's becomes of prime importance. On animal parasitology it is the most modern comprehensive and scientific work yet published in any language. We are thankful to the publisher for placing it in English before the intelligent practitioners of this country who have recently added so much to the work with which it deals. It is an erudite work and has formed the groundwork and the reference-book on protozoology and helminthology ever since its appearance. Those who have studied the subject seriously can appreciate the clearness and precision of its language, the accuracy of its information, and the value of the illustrations. To keep a work like this abreast of new knowledge the publishers would require to adopt some such methods as stop-press paragraphs or to devise the issue of supplemental insets. The editors have likewise failed to bring the edition "up to date." Their method of interpolation in the text is objectionable and confusing to the reader. This is specially noticeable at page 290, where Dr. Sambon interlards the description of the text on *Filaria bancrofti* (*Nocturna*) by some remarks about flowers and on page 291 about his priority in suggesting Dr. E. C. Low's discovery of the manner in which filaria are transmitted from the mosquito to the host. This is objectionable. On the other hand where one looks to get "up-to-date" information, as in the very important case of ankylostomes, the editor (Dr. Sambon) is silent. Why, also, is not the descriptions of the trypanosomes enlarged and brought into line with recent investigation? It is not doing Dr. Braun's book justice to fail to describe Loos's brilliant researches on the modes of the re-entry of the embryo of the ankylostomes into the host, as well as the necessary developmental changes of the exogenous phase of the embryo. The student and the practitioner in this country will look for this in Braun's book and must be disappointed to discover that he has to go elsewhere. The same may be said about tympanosomes. The only added thing is a foot-note about the *Glossina palaplis* carrying trypanosoma gambiense, the cause of sleeping sickness. This note is signed "F. V. S.," so that one does not know which of the editors to thank for the assisting note. This sort of editing is not fair to the book nor worthy of the editors. The section on the Nematodes, especially the Filariidæ, has been much extended by Dr. Sambon and is well brought up to date. It would be pleasant but for the intolerable reiterance of the questions of personal precedence which were originally quite foreign to the work. One of the best portions of Dr. Max Braun's book is the part dealing with the Trematodes (flukes or sucking-worms). The work gives an account of the anatomy, development, biology, and classification of the Trematodes and a description of those met with in man, including *Gastrodiscus hominis*, *Fasciola hepatica* (liver fluke), *Fasciolopsis buski*, *Distomum Rathausi*, *Paragomius Westermanni*, and others less common. Among the Schistosomidæ, Bilharzii hæmatobium is excellently described, and also the new one *S. Japonicum*. The interposed notes by Mr. Theobald are useful and to the point. The same may be said about the cestodes or tapeworms. A splendid account of the development of the cestode from the oncospore into the Cysticercus cellulosi in the case of the *Solium* or the C. Bovis in the case of the *Saginata*. *T. Echinococcus* so common that it lives in the liver of twenty-seven species of mammals has never been found in the human being, though its cyst has formed work for much surgery in the human liver. The volume concludes with an account of the arthropodes, which live on man, including spiders, mites, ticks, myriapods, beetles, flies, and finishes with systematic anatomical and biological remarks on mosquitoes and the biting-mouthed Brachycera—the gadflies, the Wolf-flies and Tsetse flies. One or two misprints will be corrected in the next volume, pp. 370 Argus for Argas; 406 appeares for appears; 447 Gambiene for Gambiense. This handbook should be in the library of every scientific man. The translation from the German into English has been ably done by Miss Falcke, and the book excellently printed on beautiful paper and bound in a style that will commend itself to those who must be constantly requiring help by referring to such a valuable and indispensable work.

INTERNATIONAL CLINICS. Vol. I. Sixteenth series. J. B. Lippincott Company.

THIS volume contains papers by American, English, French, and German physicians and surgeons. One by Dr. Casey Wood on poisoning by wood alcohol. This form of alcohol is sometimes drunk in America and also in

Russia, when ordinary alcoholic beverages cannot be obtained. Instances of poisoning—it may be fatal poisoning—have occurred, the most interesting feature of which is the presence of complete amaurosis after the more acute symptoms have passed away. The amaurosis may last from several days to a week or may result in permanent blindness. Apparently optic neuritis is set up which in severe cases terminates in optic atrophy. Cases of blindness due to poisoning by methyl alcohol are, however, apparently not limited to instances in which it has been drunk. Breathing in the vapour may apparently occasionally produce the same serious result, and illustrations are given of men who lost their eyesight as the result of using varnishes containing methyl alcohol. The use of such varnishes is, however, as might be expected, only dangerous when the work is carried on in a confined space with deficient ventilation. Amongst other interesting papers is one by Dr. Charles Simon on eosinophilia. Eosinophile leucocytes appear to be present around foci of infection, yet they do not appear to act as phagocytes. Neither is there evidence that they aid in the conflict with invading organisms that is taking place by discharging their granules. Dr. Simon, however, thinks that the so-called granules may be sac-like cavities in which secretion is taking place. The secretion could be discharged and the wall of the sac remaining could give the appearance of a granule. At the end of the volume is a review of the progress of medicine in its various branches.

Abbott's Alkaloidal Digest: A brief description of the therapeutics of some of the principal alkaloidal medicaments, with suggestions for their Clinical Application. By W. C. Abbott, M.D., Editor of the *American Journal of Clinical Medicine*. (Chicago: The Clinic Publishing Company. 1906.)

This small volume is dedicated to the "General Practician," who is urged by the author to stop merely "practising medicine in dull routine and to be a seeker for better ways, a real doctor, a healer of the sick." The digest is to aid him in accomplishing this most desirable acquisition by setting forth the leading therapeutic properties of the alkaloids and to give a clear conception of what is to be accomplished by these modern remedies. From the leech and the ancient lancet to the present use of osmosis and veratrine is indeed a far cry. The practitioner has to march along—not dawdle—with the therapeutic times if he is even to keep pace with the knowledge of his own clientele. This little volume will certainly make his progress more rapid, his advance easier, and his steps secure. The digest deals with "Therapeutics in brief," short therapeutic notes on single principles arranged alphabetically and gives accurate dosage and antidotes. The number of new agents of tried efficiency given here is astonishing. The doctrines advocated are the use of a single remedy for a single indication and giving the remedy in small and oft-repeated doses until the desired effect is secured. The digest further deals with the practice of alkalometry, uric acid solvents, and many other subjects of interest to medical men. The book is prepared in a very American style.

The Medical Annual, 1906. (Bristol: John Wright and Company. 7s. 6d.)

The publication of the twenty-fourth issue of this Medical Annual has been attended by difficulties caused by fire and the death of Mr. Lewis Wright by railway accident, which have caused slight delay in its issue. These being overcome, the same high standard of excellence characteristic of the work is maintained. The volume is indeed one of very exceptional interest to the practitioner. Every subject dealt with is written by experts, and no subject is omitted which is of modern interest. The Therapeutic Progress section is of particular interest, although the past year has been marked by no real advance. The Dictionary of Remedies is followed by the Dictionary of Treatment—a review of medical and surgical progress for 1905 by many contributors. The Books of the Year is a section of special interest. The surprise is that such a work can be issued at such a low price.

